# Angiogenesis-associated pathways play critical roles in neonatal sepsis outcomes

**DOI:** 10.1038/s41598-024-62195-9

**Published:** 2024-05-20

**Authors:** Mario Fidanza, Julie Hibbert, Erica Acton, Danny Harbeson, Elizna Schoeman, Patrycja Skut, Tabitha Woodman, Adrien Eynaud, Lucy Hartnell, Byron Brook, Bing Cai, Mandy Lo, Reza Falsafi, Robert E. W. Hancock, Msandeni Chiume-Kayuni, Norman Lufesi, Constantin R. Popescu, Pascal M. Lavoie, Tobias Strunk, Andrew J. Currie, Tobias R. Kollmann, Nelly Amenyogbe, Amy H. Lee

**Affiliations:** 1 Telethon Kids Institute, Perth, WA Australia; 2 Westfarmers Center of Vaccines and Infectious Diseases, Telethon Kids Institute, Perth, WA Australia; 3 Medical, Molecular and Forensic Sciences, Murdoch University, Perth, WA Australia; 4 Department of Molecular Biology and Biochemistry, Simon Fraser University, Burnaby, Canada; 5 Department of Pediatrics, University of British Columbia, Vancouver, Canada; 6 Precision Vaccines Program, Boston Children’s Hospital, Boston, MA USA; 7 Department of Pediatrics, Harvard Medical School, Boston, MA USA; 8 Department of Microbiology and Immunology, University of British Columbia, Vancouver, Canada; 9 Department of Pediatrics, Kamuzu Central Hospital, Lilongwe, Malawi; 10 Kamuzu University of Health Sciences, Lilongwe, Malawi; 11 Department of Curative and Medical Rehabilitation, Ministry of Health, Lilongwe, Malawi; 12 British Columbia Children’s Hospital Research Institute, Vancouver, Canada; 13 Department of Pediatrics, Université Laval, Québec, QC Canada; 14 Neonatal Directorate, King Edward Memorial Hospital, Perth, WA Australia; 15 Department of Microbiology & Immunology, Dalhousie University, Halifax, Canada

**Keywords:** Biomarkers, Experimental models of disease, Translational research, Immunology, Systems biology, Diseases, Pathogenesis

## Abstract

Neonatal sepsis is a major cause of childhood mortality. Limited diagnostic tools and mechanistic insights have hampered our abilities to develop prophylactic or therapeutic interventions. Biomarkers in human neonatal sepsis have been repeatedly identified as associated with dysregulation of angiopoietin signaling and altered arachidonic acid metabolism. We here provide the mechanistic evidence in support of the relevance for these observations. Angiopoetin-1 (Ang-1), which promotes vascular integrity, was decreased in blood plasma of human and murine septic newborns. In preclinical models, administration of Ang-1 provided prophylactic protection from septic death. Arachidonic acid metabolism appears to be functionally connected to Ang-1 via reactive oxygen species (ROS) with a direct role of nitric oxide (NO). Strengthening this intersection via oral administration of arachidonic acid and/or the NO donor L-arginine provided prophylactic as well as therapeutic protection from septic death while also increasing plasma Ang-1 levels among septic newborns. Our data highlight that targeting angiogenesis-associated pathways with interventions that increase Ang-1 activity directly or indirectly through ROS/eNOS provide promising avenues to prevent and/or treat severe neonatal sepsis.

## Introduction

Neonatal infection represents a significant cause of childhood mortality, potentially accounting for over a third of all global neonatal deaths^1^. Sepsis represents the most severe contributor to neonatal mortality, with 99% of global neonatal mortality occurring within low- and middle-income countries (LMICs)^2–4^. Decades of effort have resulted in only modest advances in diagnostic accuracy, clinical management, or mortality rates for neonatal sepsis^5^. Given that a causative microbial pathogen is identified in only a minority (< 30%) of sepsis cases, and with a long list of diverse causative pathogens, sole focus on targeting pathogens is unlikely to advance the field^6^. Instead progress in reducing neonatal sepsis-related morbidity and mortality could be achieved through the development of pathogen-agnostic, host-centric interventions.

The systemic immune response and dysregulated inflammation during sepsis can lead to tissue injury, organ failure and death. Irrespective of the pathogenic agent at play, endothelial dysfunction resulting in vascular leakage is the most common mechanism of injury in both neonatal and adult sepsis^7^. The Angiopoietin (Ang)/TIE-2 signaling axis is a critical regulator of vascular homeostasis and is increasingly recognized as a relevant biological mechanism underlying clinical sepsis^7–11^. Ang1 binding to the TIE-2 receptor is believed to promote vascular homeostasis and positively regulate endothelial barrier integrity^7,11^. Conversely, during periods of pathophysiological oxidative stress, Ang2 can be released from specialized weibel-palade (WP) bodies and act as a competitive antagonist for the TIE-2 receptor increasing endothelial permeability^11^. Published human data suggest Ang levels as a diagnostic biomarker and a correlate of disease severity (i.e. a prognostic biomarker) in sepsis^9,12–15^.

Furthermore, alterations in lipid metabolism and signaling pathways play an important role in the dysregulated inflammatory responses impacting the microvasculature as observed in sepsis^16^. Of note, arachidonic acid metabolism impacting most of the eicosanoid pathways has been identified as centrally important in human sepsis^17–20^. Arachidonic acid is released from the membrane phospholipids by the phospholipase A_2_ enzymes; released arachidonic acid is subsequently oxidized into proinflammatory eicosanoids by cyclooxygenase, lipoxygenase and cytochrome P450 families of enzymes^16^. We recently identified that levels of secretory phospholipase A2 type IIA (sPLA2-IIA), one of the enzymes responsible for arachidonic acid release i.e. upstream of many eicosanoid metabolites, are severely altered in neonatal sepsis and may provide a useful diagnostic biomarker^21^.

We hypothesized that beyond serving as biomarkers, insight into potential interconnection between Ang/TIE signaling and the arachidonic acid metabolism in neonatal sepsis may facilitate the identification of therapeutic or prophylactic strategies. To test this hypothesis, we assessed expression changes in the Ang/TIE and arachidonic acid signaling pathways in two human neonatal cohorts most at risk of developing neonatal sepsis: full-term infants from LMICs and premature infants from high-income countries. We then dissected associations obtained in the human studies for possible causal relationships of molecular mechanisms in a murine neonatal sepsis model amenable to experimental manipulation^22^ and found that oral administration of arachidonic acid and/or the nitric oxide donor L-arginine increased plasma Ang-1 levels which increased survival of septic newborns. These data highlight angiogenesis-associated pathways as highly promising avenues to prevent and/or treat severe neonatal sepsis.

## Results

### Ang1 and Ang2 levels are confirmed as relevant biomarkers for neonatal sepsis

**Figure 1:**
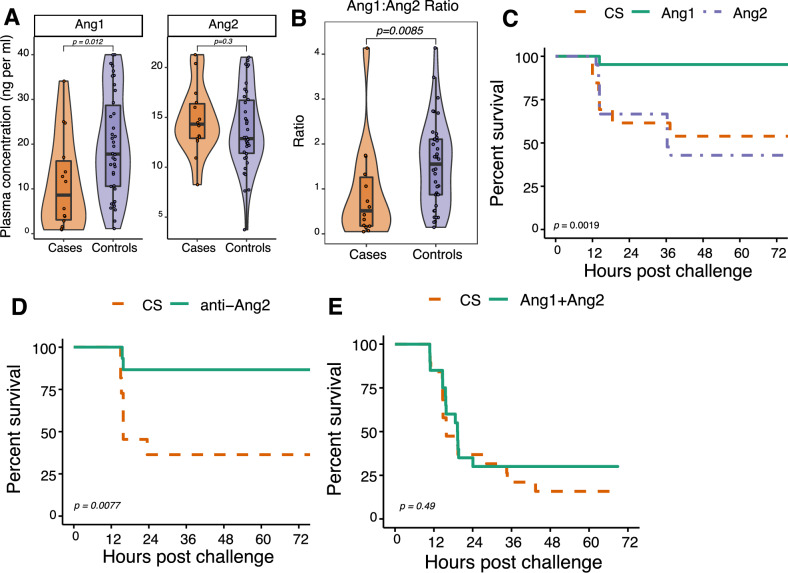
Ang/TIE signaling is associated with neonatal sepsis. (**A**) Plasma Ang1 (left panel) and Ang2 (right panel) levels in human infants with LOS (sPLA-2/Ang sepsis cohort; n = 22 infants with n = 31 samplings) or with no LOS (n = 40 infants with n = 43 samples) as measured by ELISA (Wilcoxon rank-sum with median and 95% CI). (**B**) Plasma Ang1/Ang2 ratio in human infants with LOS vs without LOS, as measured by ELISA (Wilcoxon rank-sum). (**C**) Overall survival of cecal slurry (CS)-challenged mice after prophylactic administration of exogenous Ang1 or Ang2 (n = 21 per group, derived from two independent experiments; p = 0.0019, log-rank). (**D**) Overall survival of CS-challenged mice after prophylactic administration of anti-Ang2 blocking antibody (n = 11–15 mice per group, derived from two independent experiments; p = 0.0077, log-rank). (**E**) Overall survival of CS-challenged mice treated with either saline (CS) or Ang1 plus Ang2 (Ang1 + Ang2) one hour prior to CS challenge (n = 19–20 per group, derived from two independent experiments; p = 0.493, log rank). Violin plots use a combination a boxplot and a kernel density curve to show the distribution of data. The upper and lower bounds of the boxplot indicate the first and third quartiles, respectively, with the median displayed by a horizontal line; whiskers extend no further than 1.5 × the interquartile range from the hinge.

We first sought to independently confirm the utility of Ang1 and Ang2 levels as functional biomarkers of neonatal sepsis in a cohort of human premature neonates with late-onset sepsis (LOS; onset > 72 hours of age). Relative to age-matched, non-septic controls, newborns with LOS had significantly decreased levels of plasma Ang1 (Fig. 1A, Left) and significantly elevated levels of Ang2 (Fig. 1A, Right). Additionally, the plasma Ang1/Ang2 ratio, which has previously been recognized as having prognostic significance in neonatal sepsis^7,12^, was significantly reduced in cases versus controls (Fig. 1B).

To assess whether the Ang1/Ang2 signaling pathway is relevant to sepsis outcome (i.e. survival), we used the well-controlled and standardized cecal slurry (CS) model of murine neonatal sepsis^23^. While the addition of exogenous Ang2 had little effect on survival, prophylactic Ang1 administration significantly increased survival in CS challenged mice (Fig. 1C). Neutralization of endogenous Ang2 via an antagonistic monoclonal anti-Ang2 antibody significantly improved survival in our sepsis model (Fig. 1D), and co-administration of exogenous Ang2 diminished the protective effect of exogenously administered Ang1 (Fig. 1E), indicating that Ang2 functioned as a competitive antagonist to Ang1 in our neonatal sepsis model^11^. This not only confirmed the utility of the Ang1/2 ratio as a functional biomarker of sepsis, but also suggested that Ang1 could be deployed as a prophylactic or therapeutic intervention in neonatal sepsis.

### Nitric oxide is a critical regulator of Ang/TIE signaling after cecal slurry challenge

**Figure 2:**
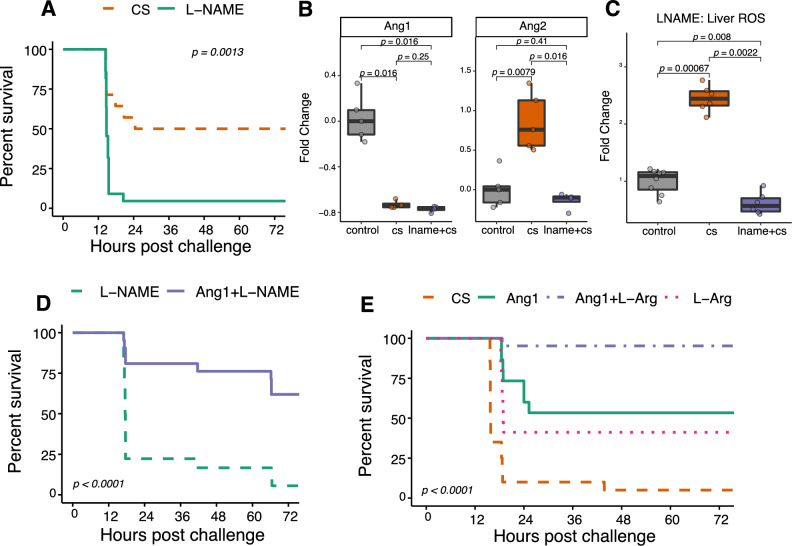
Nitric oxide regulates functionality of the Ang/TIE signaling axis. (**A**) Overall survival of CS-challenged mice with or without concurrent administration of the universal NOS inhibitor L-NAME (n = 14 CS, 22 L-NAME mice per group, derived from two independent experiments; p = 0.0013; log rank test). (**B**) Fold change over control uninfected mice of serum Ang1 and Ang2 levels in CS-challenged mice with or without concurrent administration of L-NAME (paired Wilcoxon rank-sum; n = 4–5 mice per group derived from 3 independent experiments). (**C**) Accumulation of ROS (O_2_^−^) as measured by dihydroethidium staining in the liver of CS-challenged mice with or without concurrent administration of L-NAME as measured by dihydroethidium staining (paired Wilcoxon rank-sum; n = 8 control, 6 CS, 6 L-NAME, all pooled across 3 independent experiments). (**D**) Overall survival of CS-challenged mice (at LD50) with or without administration of L-NAME 4–6 h prior to challenge (n = 18–21 mice per group, derived from two independent experiments; p < 0.0001; log rank). (**E**) Survival of CS-challenged mice (at LD90) treated with saline only, L-Arg, Ang1 or combined L-Arg and Ang1 1-h post challenge (n = 15–21 mice per group, derived from two independent experiments; *p* < 0.0001; log rank test). Boxplots indicate medians with first and third quartiles (25–75%) at the lower and upper bounds of the box; whiskers extend no further than 1.5 × interquartile range from the hinge.

Nitric oxide (NO) is a known regulator of vascular homeostasis^24^. Of its numerous biological activities, NO regulates the expression of Ang1 and the release of Ang2 from WP storage^25^. Moreover, it has also been demonstrated that endothelial nitric oxide synthase (eNOS) derived NO is required for Ang1-induced promotion of vascular integrity^26^. This suggested that NO bioavailability may be a mediator of Ang1 induced protection in our neonatal sepsis model. We first assessed the impact of the universal NOS inhibitor L-nitroarginine methyl ester (L-NAME) administered prior to CS challenge. Administration of L-NAME significantly worsened survival in our model (Fig. 2A). We next measured the effect of L-NAME on serum Ang1 and Ang2 levels. While CS challenge in the context of L-NAME induced the expected decrease in serum Ang1 levels, we observed no corresponding increase in Ang2 levels (Fig. 2B). And while the addition of L-NAME did not reduce systemic levels of exogenously administered Ang1 (data not shown), it did result in significantly reduced accumulation of reactive oxygen species (ROS) (O_2_^−^) in the livers of CS-challenged mice (Fig. 2C), suggesting that oxidative stress may lead to release of Ang2 from WP bodies^11^. In line with the mechanism of reduced NO bioavailability leading to reduced Ang1 levels, administration of Ang1 to L-NAME-treated mice prior to CS challenge reduced mortality (Fig. 2D). Given the impact of NO within our model, we considered if increasing NO bioavailability prior to CS challenge could impact survival. L-Arginine (L-Arg) is the substrate which is converted to NO by NOS. Administration of L-Arg has previously been demonstrated to modulate immune responses and increase host defense through NO-dependent mechanisms^27,28^. To allow testing of the full impact of L-Arg and Ang1 on sepsis survival we increased the challenge dose to a lethal dose (LD) 90. Administration of L-Arg 1h post challenge alone but especially in combination with Ang1 significantly improved survival in our neonatal sepsis model (Fig. 2E).

### Arachidonic acid signaling impacts on angiogenesis-associated pathways

#### Gene expression changes identify arachidonic acid metabolism as a key regulator of outcome in a mouse neonatal sepsis model

Our data indicated that administering L-Arg alone and especially together with Ang1 could potentially serve as an effective therapeutic intervention to reduce death from sepsis. While endothelial disruption and subsequent vascular leakage represent a critical step in sepsis that could be addressed via Ang1 +/− L-Arg therapy, the functional pathways that destine some individuals, but not others, to succumb to sepsis have not been delineated. This precludes targeting possible prophylactic or early therapeutic interventions to those at highest risk of death from sepsis. We thus set out to identify possible risk-associated molecular mechanisms using RNA-seq conducted on samples taken at 18 hours post an LD50 challenge in a mouse neonatal sepsis model.

**Figure 3:**
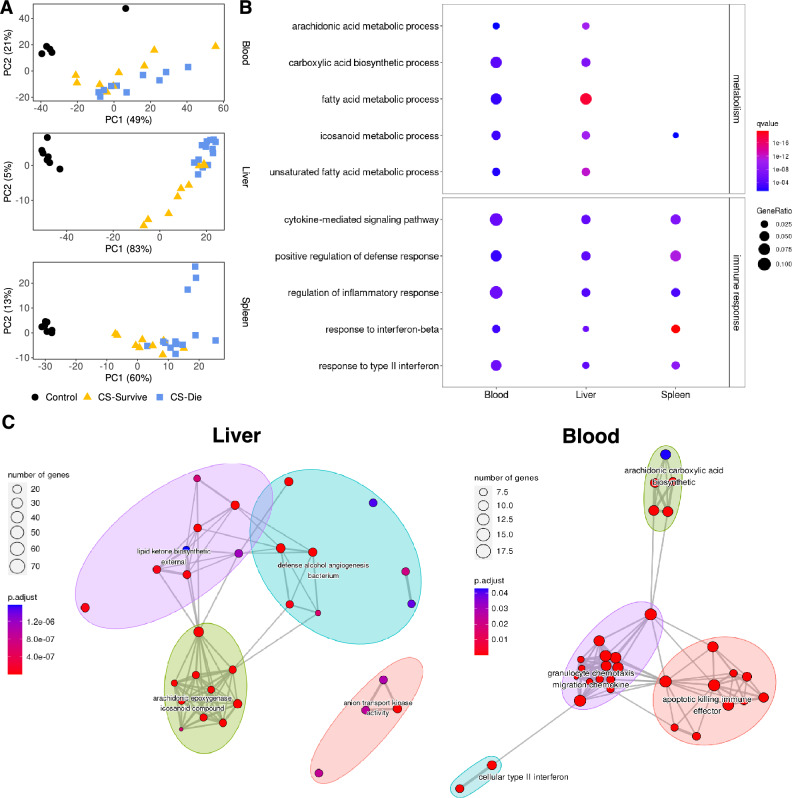
Whole blood, liver and spleen transcriptomics comparing likely survivors and non-survivors of murine neonatal sepsis. (**A**) Unsupervised clustering of normalized RNA-seq data using principal component analyses of transcriptomic data collected from blood ^1^, liver (middle) and spleen (bottom); (n = 7 Control, 9 CS-Survive, 10 (blood) or 13 (liver, spleen) CS-Die; derived from 5 independent experiments). (**B**) Selected immune response and metabolic pathways from top enriched GO terms (by q-value) from differentially expressed genes in predicted survivors and non-survivors in the blood, liver, and spleen. (**C**) Network of clustered enriched GO biological process terms from differentially expressed genes in predicted survivor vs non-survivor mice in the liver and whole blood. The size of the node reflects the number of genes in the term, while the colour represents the adjusted *p*-value. Each functional cluster is represented by a different color. Edges connect terms that have shared genes (similarity > 0.2), and the length of the edge is inversely proportional to term similarity.

All CS-challenged neonatal mice were classified into likely survivors or non-survivors using a gradient boosting machine learning model based on physiological and behavioral animal health scores as we published previously^23^. Specifically, a series of machine learning models were trained on a cohort of 222 CS-challenged pups with known outcomes and tested on a random selection of 74 pups. The final gradient boosting machine model was able to accurately classify pups as likely survivors or likely non-survivors (accuracy score of 0.85; Table S1)^23^. Whole blood, liver, and spleen samples were collected from neonatal mice following CS or sham challenge (controls). Liver and spleen were selected in addition to blood to help uncover the impact of sepsis on metabolic and hematopoietic processes. Unsupervised principal component analyses on the normalized RNA-seq data across the top 500 most variable genes revealed a clear separation not only between challenged and unchallenged mice but also between likely survivors and non-survivors (Fig. 3A). Challenged mice exhibited expression patterns clearly distinct from healthy control mice. While challenged mice were generally more similar to each other, likely representing that all pups were in the septic state, pups predicted to survive still separated from pups predicted to die (Fig. 3A, for complete list of differentially expressed genes see Supplementary Data 1). Gene Ontology^4^ functional enrichment analysis of the differentially expressed genes between survivors and non-survivors for each tissue revealed common as well as tissue-specific enriched terms across the blood, liver, and spleen (Fig. 3B, Fig. S1). After creating a similarity matrix using the overlap of GO term enrichments (see Methods), the top 5 clusters of terms were selected to evaluate the major biological processes enriched for each tissue (Fig. 3C). From these, the top 15 most enriched terms per tissue by q-value centered around the immune response (all tissues) and metabolic processes (the blood and liver) (Fig. S1). A subset of these pathways is shown for clarity in Fig. 3B, in which arachidonic acid metabolism was enriched as differentially regulated between survivors and non-survivors in both liver and blood (Fig. 3C). Low levels of AA have been associated with alterations of various immune cell subsets and an increased risk for late-onset sepsis in premature human infants^17,29^. Considering the broad scope of the AA-related genes driving the observed GO enrichment, as well as the overall prominence of these signatures across all three tissues in the differentiation of risk for death between predicted survivors and non-survivors, we hypothesized that AA levels may play a role in determining the risk for death in our sepsis model by regulating Ang/TIE signaling.

#### Arachidonic acid metabolism modulates Ang/TIE signaling in a mouse model and may be relevant in human neonatal sepsis

**Figure 4:**
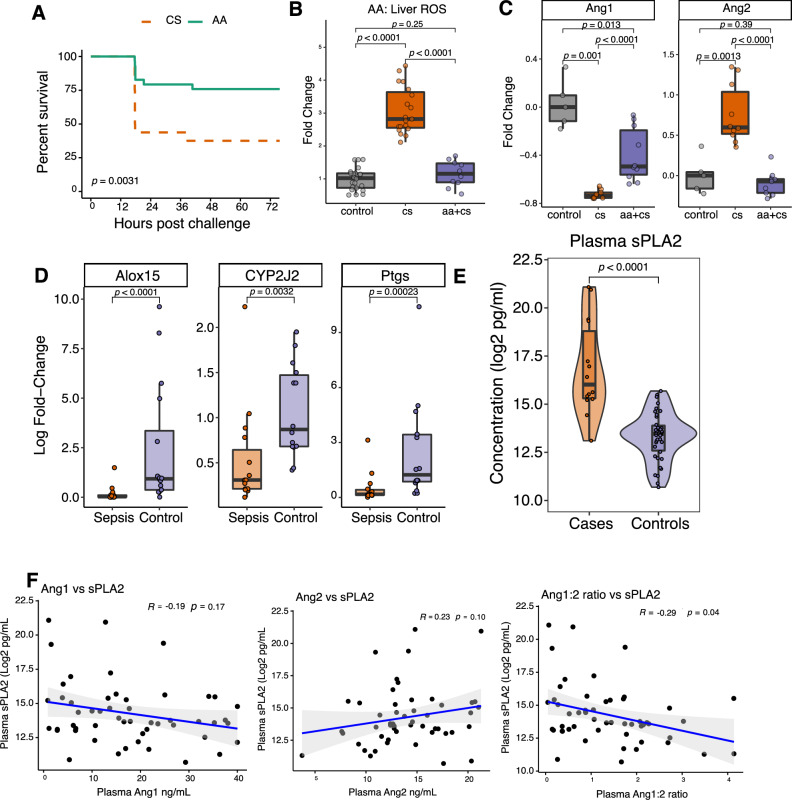
Arachidonic acid signaling modulates Ang1/Ang2 ratio and is relevant in murine and human neonatal sepsis. (**A**) Overall survival of CS-challenged mice with or without prophylactic AA treatment 4–6 h prior to challenge (n = 16 CS, 29 AA mice, derived from two independent experiments; *p* = 0.003, log rank). (**B**) ROS (O_2_^−^) accumulation in the liver of CS-challenged mice 8 h post challenge as determined by relative fluorescence intensity of dihydroethidium normalized to untreated controls (p < 0.001, paired Wilcoxon rank-sum test, n = 22 controls, 19 cs, and 10 AA treated mice (CS + AA) derived from 3 independent experiments). (**C**) Serum Ang1 and Ang2 levels in control, challenged (CS) and challenged / AA treated mice (CS + AA) (paired Wilcoxon rank-sum; n = 5 control, 10 cs, and 10 AA treated mice (CS + AA) derived from 3 independent experiments). (**D**) qPCR analysis of fold changes between *ALOX15* (left), *PTGS2* (middle) or *CYP2J2* (right) to reference gene *TUBB* in a cohort of septic neonates from Malawi (n = 14 cases and 16 controls; Wilcoxon rank-sum). (**E**) Plasma sPLA-2 levels in human infants with any LOS vs. no LOS, as measured by ELISA (Wilcoxon rank-sum). (**F**) Spearman correlation showing relationship between plasma sPLA-2 levels and plasma ANG1 levels (left-panel), plasma ANG2 levels (middle panel) and plasma ANG1/ANG2 ratio (right panel) for all neonates (22 cases and 40 controls) within our cohort (Spearman correlation). Boxplots indicate medians with first and third quartiles (25–75%) indicated by the lower and upper bounds of the box; whiskers extend no further than 1.5 × interquartile range from the hinge. Violin plots display a kernel density curve overlaying a boxplot.

To assess the impact of AA metabolism on Ang/TIE signaling in the murine neonatal sepsis model, we administered AA (2mg) by oral gavage 4–6 hours prior to CS challenge as described^30^. Exogenous AA significantly improved survival when compared to vehicle control (Fig. 4A). The mechanisms underlying the association between low AA levels and increased risk for sepsis are not known. The inflammatory response inherent to sepsis results in pathophysiological production and accumulation of ROS, and oxidative stress-induced injury can play an important role in the development of end-organ failure in sepsis^18^. Of the numerous effects of ROS on cells, one of the best described is the oxidation of membrane fatty acids, which has deleterious effects on cell signaling and protein function^19^. Though AA is an important precursor to lipoxins and other important resolvins, AA itself has been implicated in the defense against oxidative stress via the induction of cellular antioxidants^31^. We thus hypothesized that AA-induced protection from sepsis in our model may be mediated through a reduction in oxidative stress. As the liver exhibited the greatest number of differentially expressed genes related to oxidative stress, we administered AA prior to CS challenge and utilized dihydroethidium to measure ROS (O_2_^−^) accumulation in the liver post challenge. AA administration induced a significant reduction in ROS accumulation relative to sham-treated, CS-challenged controls. Specifically, prophylactic administration of AA reduced ROS accumulation in the liver to levels similar to that of uninfected control mice (Fig. 4B).

Both eicosanoids and oxidative stress are known critical regulators of angiogenesis^32,33^. GO term enrichment of our data had highlighted vascular processes and angiogenesis as relevant to survival (Fig. 3C). While AA metabolism had not been previously linked to the Ang/TIE-2 axis, a functional interaction between these two pathways was suggested by the finding that mutations in both have independently been linked to a rare form of retinopathy^34,35^. To assess the impact of AA administration on Ang1/TIE-2 pathways in the mouse neonatal sepsis model, we used enzyme-linked immunosorbent assays (ELISA) to assess serum levels of Ang1 and Ang2 post CS challenge and in relation to AA administration. CS challenge induced a significant increase in serum Ang2 and a corresponding decrease in Ang1 levels relative to unchallenged controls; prophylactic administration of AA prior to CS challenge completely abrogated the CS-induced increase in serum Ang2 levels and significantly reduced the magnitude of the CS-induced reduction in Ang1 (Fig. 4C).

To assess if the altered AA metabolism we identified in our murine model was also altered in human neonatal sepsis, we evaluated the expression of genes central to the primary AA metabolic pathways in neonates with sepsis. AA metabolism can broadly be separated into the cyclooxygenase pathway and the lipoxygenase pathway; together these pathways give rise to 4 primary classes of eicosanoid metabolites, many of which regulate both angiogenesis and vascular integrity^31,32^*.* We found that relative to uninfected controls, human neonates with clinical sepsis exhibited significantly reduced expression of *ALOX15, PTGS2* and *CYP2J2,* implicating all main AA metabolic pathways in sepsis (Fig. 4D)*.* The dysregulation of multiple AA metabolism genes in neonates with sepsis suggests wide-spread perturbation of AA metabolism in septic newborns, potentially originating upstream of the genes we had measured. One of the primary functions of sPLA-2 is to catalyze the membrane release of AA^36^*.* Considering its role as an upstream regulator of both AA metabolism and mobilization, dysregulation of sPLA-2 could serve as an upstream indicator and biomarker of wide-spread downstream disruption across several pathways of AA metabolism. We had previously shown that sPLA-2 levels have clinical utility in the early diagnosis of LOS in very preterm infants^21^. Within the same cohort previously assessed for sPLA-2 levels in association with sepsis, we showed that sPLA-2 levels are lower in very preterm infants with LOS compared to those without (Fig. 4E). Furthermore, we identified a direct quantitative relationship between plasma sPLA-2, ANG1 and ANG2 levels that was irrespective of sepsis status. The plasma ANG1/ANG2 ratio was significantly negatively correlated with sPLA-2, while plasma Ang-1 and Ang-2 trended towards negative and positive correlations respectively for the Australian cohort of 22 sepsis cases and 40 controls (Fig. S2), further supporting an association between AA metabolism and the ANG1/ANG2 axis of vascular integrity (Fig. 4F).

## Discussion

**Figure 5:**
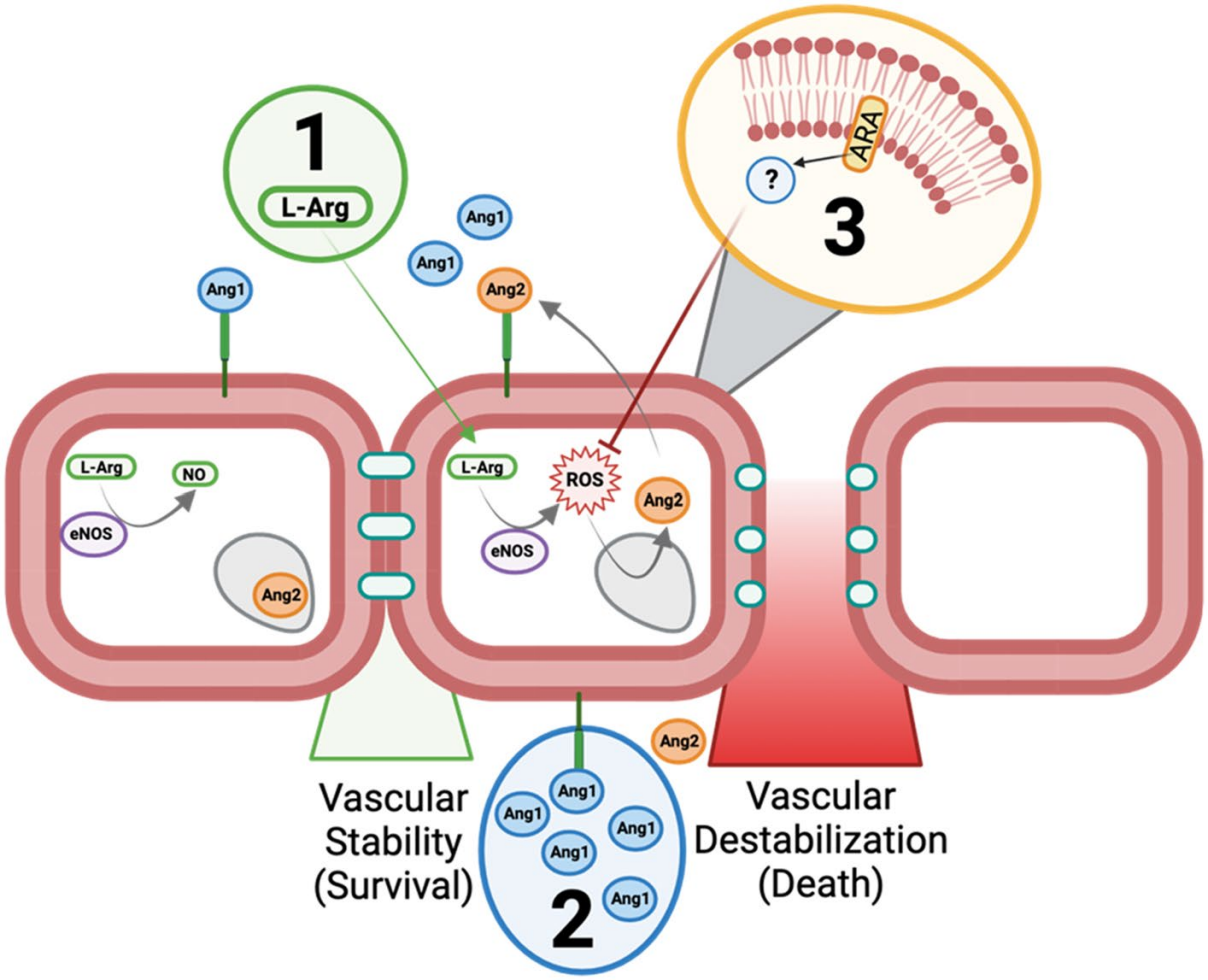
Proposed model of enhancing vascular resilience in neonatal sepsis via 3 complementary strategies. During the steady state, vascular integrity is maintained by sequestration of Ang2 within intracellular vesicles. eNOS remains coupled to the cellular membrane, and converts L-Arg into NO. During the septic inflammatory response, eNOS is uncoupled from the cellular membrane and converts L-Arg into ROS. ROS releases Ang2 from within the endothelial cell which outcompetes Ang1 from binding to the TIE2 receptor. The Ang2:TIE2 complex triggers the release of tight junctions and causes vascular instability. Vascular resilience was enhanced through three avenues: (**1**) L-Arg supplementation ensured that eNOS could access sufficient substrate to generate NO instead of ROS, improving survival outcomes. (**2**) Exogenous Ang1 ensures a more favorable Ang1:Ang2 ratio which led to decreased levels of ROS improving survival. (**3**) Oral supplementation with ARA prior to challenge resulted in decreased levels of ROS in the liver during sepsis (via a still unknown mechanism), while increasing levels of plasma Ang1 and improving survival outcomes. *L-Arg* L-arginine, *NO* nitric oxide, *eNOS* endothelial nitric oxide synthase, *Ang1* angiopoetin-1, *Ang2* angiopoetin-2, *ROS* reactive oxygen species, *ARA* arachidonic acid. This image was created using Biorender.

Vascular dysfunction is a recognized hallmark that underlies the pathophysiology of multi-system organ failure in sepsis^37^. Developing a better understanding of the biological mechanisms that contribute to this dysfunction, particularly in the neonatal setting, offers translational potential for the development of universal, pathogen-agnostic, host-centered prophylactic or therapeutic modalities^15^. The data we present here indicate that dysregulation of angiopoietin signaling during neonatal sepsis is functionally connected to AA signaling via ROS and NO. By targeting the angiopoietin axis from three nodes both upstream and downstream of Ang signaling, we demonstrated that survival outcomes could be improved, coinciding with increased Ang1 plasma levels (Fig. 5). Beyond this novel molecular mechanistic insight, both AA and L-Arg are feasible oral interventions to be explored in the prevention or treatment of neonatal sepsis.

Low levels of plasma AA in premature infants are associated with an increased risk of LOS, but little is known about the biological mechanisms that underlie this association^17,21^. In the context of infection, most of the focus on eicosanoids has centered on their anti-inflammatory activities; however they also play a critical role in regulating vascular homeostasis and angiogenesis^33,38^. Our results provide evidence of interconnection between AA and the Ang/TIE signaling axis during the septic inflammatory response. Our data further reveal that this effect was mediated through the AA-induced reduction in ROS accumulation during sepsis which can be responsible for inducing the release of Ang2 from WP bodies. We did not identify the mechanism by which AA supplementations directly reduces ROS levels in the liver or modulates serum levels of Ang-1 or Ang-2, as indicated by the question mark in Fig. 5. While the relevance of the Ang/TIE signaling axis to the pathophysiological progression of neonatal sepsis has been reported previously, this work establishes a link between AA metabolism and the regulation of the Ang/TIE signaling pathway. Our findings further support the notion that the Ang1/Ang2 ratio along with plasma sPLA-2 concentrations may hold diagnostic and/or prognostic value in neonatal sepsis^3,7,21^. Advancing this insight, the functional intersection of these two pathways we identified here offers feasible translational avenues including supplementation to high-risk neonates. AA and/or L-Arg supplements can be administered orally, which is especially relevant in resource poor settings, where most cases of neonatal sepsis occur^1^.

The mechanistic results derived from the murine CS challenge model highlight the important role of NO in homeostatic Ang/TIE signaling. The universal nitric oxide synthase inhibitor L-NAME dramatically reduced survival in our model. Prophylactic administration of Ang1 reduced the detrimental impact of L-NAME, revealing that this influence, irrespective of the upstream enzyme generating NO, was largely related to a downstream disruption of the Ang/TIE signaling network. Administration of L-NAME also resulted in a significant decrease in ROS accumulation in the livers of CS-challenged mice. It has previously been demonstrated that uncoupled eNOS is a significant source of superoxide free radicals; the production of which is typically inhibited by the presence of L-NAME^39,40^. Uncoupling of eNOS and corresponding production of O_2_^−^ is also directly augmented by C-reactive protein, a diagnostic marker often used to guide management of clinical sepsis^41^. Interestingly, while administration of L-NAME immediately prior to challenge did not impact the sepsis-induced reduction in serum Ang1 levels, the corresponding increase in serum Ang2 concentration was completely absent in challenged mice that received L-NAME. This may indicate that while oxidative stress does not directly impact the regulation of Ang1 expression, release of Ang2 from WP bodies is dependent on ROS accumulation. Future work will have to decipher the exact sequence of events.

Our study has limitations. Firstly, we were unable to differentiate between NO derived from eNOS versus that derived from iNOS from sources such as macrophage oxidative burst. Considering the many and complex roles of NO for sepsis progression, the choice to interrogate the impact of NO in our model as broadly as possible with L-NAME was intentional, as we wished to assess the direct impact of NO on the Ang/TIE signaling axis. Our data do not exclude a role of NO distinct from impacting AA, thus contribution of NO derived from eNOS versus iNOS on Ang1/2 levels and with that on sepsis progression, and the temporal dynamics of each of these sources, clearly merits further investigation. We did not delineate the exact eicosanoid molecule/s that mediate the impact on NO and with that Ang-Tie2 signaling. But as perturbation of all eicosanoid pathways in both of our human and mouse neonatal sepsis data indicated a broad functional interconnection across multiple pathways, identification of individual molecules was of lower priority. This of course is important and will need to be delineated in future work. Furthermore, we did not define the precise pharmacological parameters of AA, L-Arg or Ang1 administration in relation to optimal prophylactic or therapeutic interventions nor their interaction, and thus have not identified key parameters such as effective ratios or thresholds for each; this will require careful time and dose assessment in future studies. Additionally, although altered AA metabolism appeared as relevant in both clinical contexts, we would not be able to directly contrast the dynamics of AA metabolism or Ang/TIE signaling between our term and preterm human neonatal cohorts. Further investigation is required to understand how gestational age at birth impacts the mechanisms and associations identified in our study. For example, AA accumulation begins in earnest during the third trimester of gestation and continues until birth; premature infants are therefore often AA deficient relative to term neonates^42–44^. Additionally, specific morbidities related to premature birth such as retinopathy of prematurity are associated with dysregulation of the Ang/TIE signaling axis^34^. Existing evidence thus suggests that discrete aspects of both AA metabolism and Ang/TIE signaling may differ in premature versus term neonates; this will have to be addressed in targeted studies.

Our data provide a functional link between AA metabolism and regulation of the Ang/TIE signaling axis during sepsis. The validation of our findings from the controlled murine model in human cohorts of septic neonates highlights the potential this signaling axis holds for clinical diagnostic and prognostic purposes. The feasibility of human interventions (AA, L-Arg, Ang1) that in our murine model have proven effective both as a prophylactic and a therapeutic intervention, thus offering avenues that clinical trials targeting this signaling axis could now explore for the development of effective pathogen-agnostic, host-centered preventions or therapeutics for neonatal sepsis.

## Methods

### Mice and monitoring

All animal work conducted was approved by the Animal Ethics Committee at Telethon Kid’s Institute for mechanistic work (AEC351, AEC363) and the University of British Columbia for transcriptomic studies (A17-0110). Specific pathogen-free mouse breading pairs for C57BL/6J mice were purchased from The Jackson Laboratory and bred in-house. To generate neonatal mice, paired matings were established weekly, females were isolated from males after being visually identified as pregnant and then monitored daily thereafter to ensure an accurate date of birth was determined for all experimental mice. Both male and female mouse pups were used throughout all animal experiments. All experimental mice were monitored as previously described^23^. Briefly, mice were monitored every 8 to 12 hours for the first 2 days post challenge and then daily thereafter. Humane end-point was determined using a righting reflex and mobility score as previously described^23^.

### Murine neonatal sepsis challenge

The neonatal sepsis model was utilized as previously described. In short, CS was obtained from adult male caeca and resuspended in dextrose 5% water at a concentration of 100 mg/mL and then passed through a 70 m filter. To provide consistency, our cecal slurry model^22^ employs multiple litters of adult donors of cecal slurry that are pooled, mixed and aliquots were frozen at −80°C until challenge. Following this, each slurry is titred to determine the effective dose (LD_50_ etc.). Each new cecal slurry lot is directly compared to previous lots to ensure consistency across experiments. Neonatal mice at day of life (DOL) 6–8 were challenged via intraperitoneal injection at a weight adjusted dose of 1mg/g body weight. Litter-to-litter variation was accounted for by having a balanced number of treated and control mice in each litter.

### Transcriptomics and statistical analyses

Total RNA was extracted from each sample using the RiboPure RNA purification kit (Ambion ThermoFisher; Waltham, MA, USA) following the manufacturer’s protocol. Quantification and quality assessment of total RNA was performed using an Agilent 2100 Bioanalyzer^34^. Poly-adenylated RNA was captured using the NEBNext Poly(A) mRNA Magnetic Isolation Module (catalog no.: E7409L, NEB). Strand-specific cDNA libraries were generated from poly-adenylated RNA using the KAPA Stranded RNA-Seq Library Preparation Kit (cat. no.: 07277253001, Roche). All cDNA libraries were prepared at the same time and sequenced on the HiSeq 2500 (Illumina). Sequence quality was assessed using FastQC v0.11.5 and MultiQC v0.8. The FASTQ sequence reads were aligned to the mouse genome (Ensembl GRCm38.94) using STAR v2.5 and mapped to Ensembl transcripts. Read-counts were generated using htseq-count (HTSeq 0.6.1p1). All statistical analysis and figure generation was performed in R v4.3.1. RNA samples with < 1 million reads after globin removal were excluded from analysis. Non-challenged mice with indicators of sickness such as weight loss or high CFU in the blood, liver, or spleen were removed from analysis, as were challenged mice that gained weight. One challenged mouse was removed due to sacrifice at 18 hours rather than 24 hours. Normalization and generation of DE gene lists was done with DESeq2 v1.40.2—genes were considered to be differentially expressed with a Benjamini–Hochberg adjusted p-value of < 0.05 and a FC value of ± 1.5. GO term biological process functional enrichment analysis was performed on genes differentially expressed between survivors and non-survivors for each tissue with clusterProfiler v4.8.2^45^, and filtered with a q-value of < 0.05. A similarity matrix of terms based on overlapping gene sets (using Jaccard coefficients) from each enrichment output was created and the top 5 GO term clusters for each tissue were selected and plotted using enrichplot v1.20.0^46^.

### Survival experiments

For prophylactic AA experiments, 2 mg of AA (Fisher Scientific: #ICN19462510) was diluted in 30 μL of corn oil (Sigma C8267) which was administered 4–6 hours prior to CS challenge via oral gavage. Control animals received 30 μL corn oil alone. All other experiments used saline controls. For exogenous Ang1 and Ang2 experiments, 500 ng of Ang1 or Ang2 (R&D Systems: #9936-AN and #7186-AN respectively) was administered via intraperitoneal injection 1 hour prior to CS challenge. For anti-Angiopoietin-2 antibody treatment 50 μg of antibody (Adipogen: #AG-27B-0016PF) was administered concurrently with CS challenge via intraperitoneal injection. For L-NAME experiments, 10 μg of L-NAME (Abcam: #120136) reconstituted in distilled water was injected via intraperitoneal injection concurrently with CS challenge. Finally, for L-Arginine experiments, 5 mg of L-Arginine (Cayman Chemical: #23703) reconstituted in distilled water was administered via intraperitoneal injection 4-6 hours prior to CS challenge. The schedules described above were maintained for all combinational interventions described.

### ROS quantification

For ROS detection, mice were treated according to the schedule described above. Eight hours post CS challenge we administered dihydroethidium (DHE) (Cayman Chemical: #12013) reconstituted in DMSO at a concentration of 10 μg/g body weight via intraperitoneal injection. After a 20 minute incubation period, mice were euthanized, and livers were excised and immediately drop fixed in 4% paraformaldehyde (Thermo Scientific: #AAJ19943K2) for 48 hours. Livers were then snap frozen, sectioned and DAPI counterstained. DHE was visualized using fluorescence microscopy utilizing a standard ethidium bromide filter. Relative ROS quantification was performed using ImageJ as previously described^36^. Briefly, a DAPI counter-mask was created to isolate nuclei, background fluorescence was subtracted, and MFI of DHE was calculated. All results are normalized relative to batch-matched unchallenged controls that were imaged under identical parameters.

### Murine angiopoietin 1 and 2 ELISA

Mice were treated according to the above-described schedule. Blood was drawn 2 or 4 hours post CS challenge via cardiac puncture. Blood was allowed to clot at room temperature for 15 minutes at room temperature prior to centrifugation to isolate serum. Mouse Ang1 (Sapphire Biosciences: #LS-F2956) and Ang2 (R&D Systems: #MANG20) ELISAs were performed according to manufacturer instructions.

### Human study (sPLA-2/Ang cohort) participants and whole blood sampling

A total of 87 infants were enrolled in a prospective, observational study that was approved by the institutional ethics review board at King Edward Memorial Hospital (RGS0000000862)^21^. Blood plasma samples were collected near the time of blood culture sampling for suspected LOS (± median 1.2 (IQR 25th–75th 0.0–7.5) hours), and samples were stored at −80°C until analysis. ‘Any LOS’ was defined as a positive blood culture and/or a CRP of ≥ 20 mg/L within 72 hours of culture sampling^38,39^. Suspected sepsis episodes with a negative blood culture and 2–4 serial CRP < 20 mg/L within 72 hours of culture sampling were defined as ‘no LOS’. Infants were assigned an overall LOS classification based on the most severe outcome of all episodes (Fig. S3). Three suspected LOS episodes with a positive Gram-positive coagulase-negative staphylococci blood culture, 2–4 serial CRP < 20 mg/L within 72 hours of blood culture sampling, and the absence of sustained clinical features of LOS were classified as blood culture contaminants were excluded, as were episodes of necrotizing enterocolitis (n = 3).

The basic demographic of the 46 extremely premature infants included in the analysis were similar between the any LOS (n = 15) and no LOS infants (n = 41), except any LOS infants were older at the time of blood culture sampling (Table S2). Two infants in the LOS group died from multi-organ failure septicaemia and ten infants (any LOS n = 5; no LOS n = 5) died from causes unrelated to LOS; all data collected prior to death were included in the analysis. We performed analyses using the first LOS sample for each individual to minimize bias (Table S2).

### Human sPLA-2, Ang1 and Ang2 ELISA

Plasma sPLA-2 Type IIA, Ang1 and Ang2 was measured by ELISA kit (Cayman Chemical: #501380; R&D Systems: #DANG10, #DANG20, respectively), as per manufacturer instructions. Samples were run in duplicate with absorbance read at 450 nm in real time using a spectrophotometer. sPLA-2, Ang1 and Ang concentrations were generated from a seven-point four-parameter logistic standard curve.

### Malawi cohort – qPCR

Gene expression data was generated from samples obtained from young infants under three months of age who presented with suspected sepsis (as per the receiving clinician) at the kamuzu central hospital (KCH) in Lilongwe, Malawi^47^. In the parent study, whole blood was collected from infants with suspected sepsis at the time of blood culture sampling in RNALater (Ambion/Invitrogen RNALater tubes, catalog number AM7022) along with matched non-septic controls admitted to KCH without any signs or symptoms of sepsis. The samples from this cohort were collected for the descripted purpose, under ethics approval from the National Health Sciences Research Committee (#17/8/1819) and the University of British Columbia Children’s & Women’s Research Ethics Board (#H16-02639). Samples were stored at −80°C for batch analyses. After thawing on ice, samples were centrifuged at 14.000 rpm for 4 minutes. Plasma was used for metabolomics analyses, whereas cell pellets were used for gene expression analyses. RNA was extracted from cell pellets using the RiboPure RNA Purification Kit (catalog number AM1928). Quantification and quality assessment of total RNA was performed on an Agilent 2100 Bioanalyzer. PCR was subsequently carried out for the genes of interests. Expression of *ALOX15*, *PTGS2*, *CYP2J2*, and *TUBB* (housekeeping gene) were compared for 16 control samples (i.e. infants admitted to the neonatal unit for a variety of conditions such as mild prematurity, jaundice, low birth weight, and for whom antibiotics were not indicated or administered) and 14 sepsis samples of the neonatal cohort (Table S3) by qPCR. Infants were matched by biological variables known to be associated with neonatal sepsis including: sex, gestational age and birth weight (Table S3). qPCR primers were designed to amplify transcripts such that either the forward or reverse primer bridged an exon-exon junction. Primers were designed using Primer-BLAST^41^ for the primary transcript. If more than one transcript was of interest, common exons were chosen for the primer pairs. The product size was set between 100–250 bp and the primer set had to span an exon-exon junction (otherwise, separate exons were used for the primer pair). All primer pairs were specific to the transcripts of interest. The location of the primers were verified using the CCDS of the specific gene and the Reverse Complement tool^48^.

### Statistical analysis

For mouse survival experiments, survival curves were compared between groups using a long-rank test. Generally, data were tested for normality either by using the Shapiro-Wilk test, rejecting the null hypothesis when *p* < 0.1, or through visual inspection of qqplot and distribution. For ELISAs, ROS analysis, and qPCR, paired Wilcoxon rank-sum or Student’s *t* test were applied with adjustment for multiple comparisons (via Benjamini-Hochberg (BH) adjustment) when applicable. sPLA2 and ANG1/ANG2 correlations were assessed by Spearman’s correlation coefficient with corresponding *p* value reported within the relevant figure panel. Differences were considered significant with p < 0.05 and exact *p* values are presented in relevant figure legends.

### Ethics approval

All animal work conducted was approved by the Animal Ethics Committee at Telethon Kid’s Institute for mechanistic work (AEC351, AEC363) and the University of British Columbia for transcriptomic studies (A17-0110). All animal work performed at UBC was in line with the Canadian Council on Animal Care, and at Telethon Kids, the Australian & New Zealand Council for the Care of Animals in Research and Teaching (ANZCCART). All animals were euthanized using methods approved by the CCAC or ANZCAART. We followed the ARRIVE guidelines for animal work as outlined in our methods, including: both sexes were used, mouse strain designation, censorship, randomization schemes and endpoint criteria for survival experiments were all clearly described.

Samples from the preterm Australian cohort was approved by the institutional ethics review board at King Edward Memorial Hospital (RGS0000000862). Samples from the term Malawi cohort were collected for the descripted purpose, under ethics approval from the National Health Sciences Research Committee (#17/8/1819) and the University of British Columbia Children’s & Women’s Research Ethics Board (#H16-02639). Written parental consent were obtained for both human studies.

### Supplementary Information

Supplementary Information 1.

Supplementary Information 2.

## Data Availability

The count files are deposited under GEO accession number GSE239431. The raw fastq files can be downloaded from SRA under the BioProject PRJNA999296. Analytical codes used to produce Figures 1 to 5 are publicly available via GitHub under the URL https://github.com/ImmuneResilience/ang_neosepsis.
