# Supplementary material for: Angiogenesis-associated pathways play critical roles in neonatal sepsis outcomes
Source: Sci Rep. 2024 May 20;14:11444. doi: 10.1038/s41598-024-62195-9 (PMC11106288; doi:10.1038/s41598-024-62195-9)
Supplement: Supplementary file 1 — Supplementary Information 1. [file 41598_2024_62195_MOESM1_ESM.docx]

**Supplementary Figures**

**
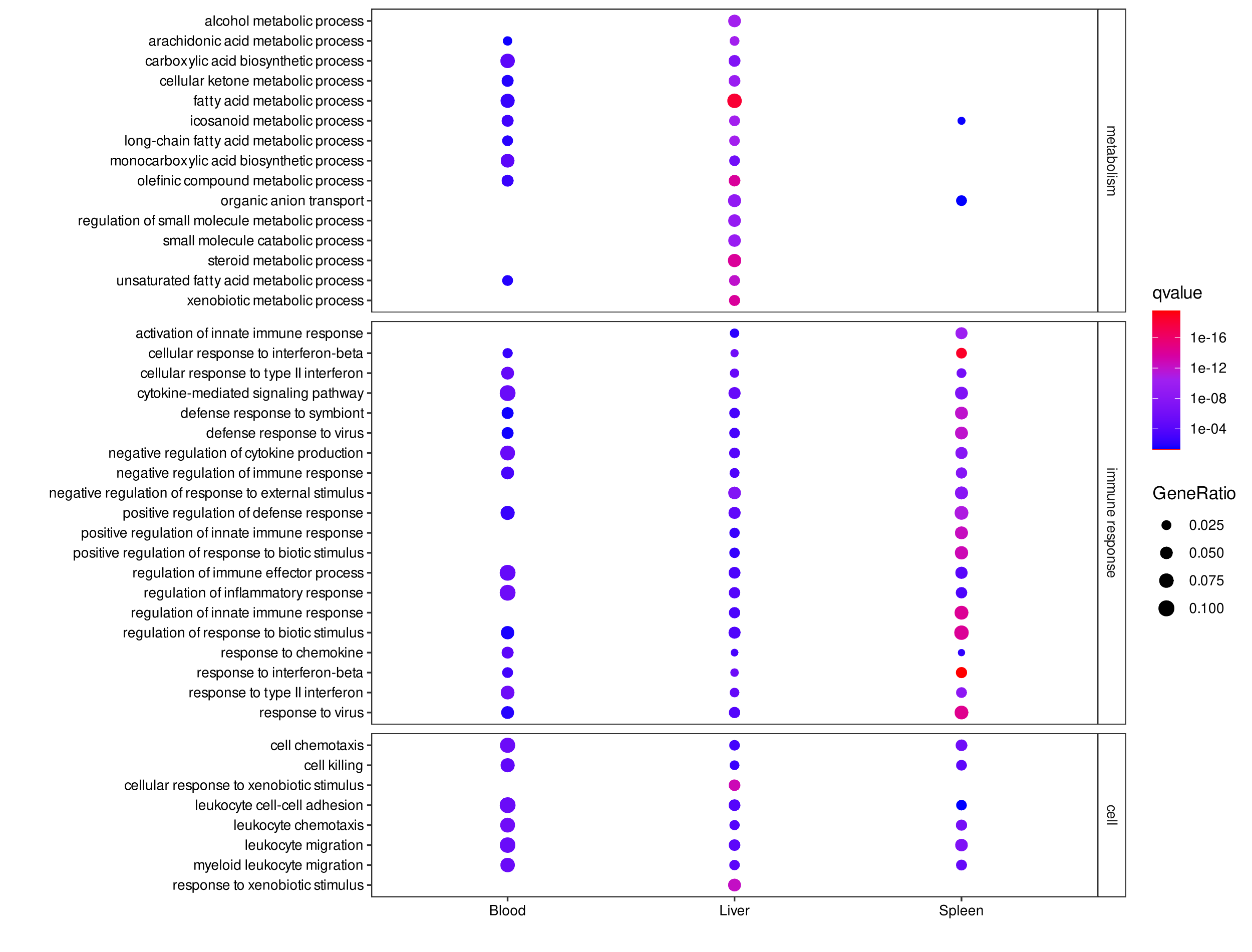
**

**Figure S1.** Top 15 enriched GO terms representing differences between survivors and non-survivors in the blood, liver, and spleen

**Figure S2. Sample inclusion / exclusion criteria for sPLA-2/Ang human cohort.**

**Supplementary Tables**

| **True class** |  |  |
| --- | --- | --- |
| Survivor | **33** | 6 |
| Non-survivor | 5 | **30** |
|  | Survivor | Non-Survivor |
|  | **Predicted class** | |

**Table S1. Confusion matrix showing accuracy of the Gradient Boosting Machine model.** Using monitoring data collected at 18 and 24-hours post challenge (weight, righting reflex and mobility), a Gradient Boosting machine learning model was able to distinguish survivors and non-survivors with an accuracy score of 0.85; 33/39 and 30/35 non-survivors were correctly classified. The 95 % CI for accuracy was 0.784-0.932 using 1000 bootstrapping iterations to assess model performance. Specifically the GradientBoostingClassifier from sklearn.ensembl was run with the minimum samples per leaf set to 2 and number of estimators = 100.

| Feature | Median (interquartile Range) or Number (%) | | |
| --- | --- | --- | --- |
|  | **First episode of LOS**  **(Infants n=15)** | **No LOS**  **(Infants n=41)** | ***p* value** |
| Gestational age (weeks) | 24.7 (23.9 – 27.0) | 25.7 (24.0 - 27.1) | 0.815 |
| Birthweight (grams) | 760 (600 - 835) | 730 (565 - 965) | 0.830 |
| Male | 8 (53.3%) | 21 (51.2%) | 1.000 |
| Postnatal age (days) | 9 (6 - 39) | 9 (6 - 20) | 0.731 |
| Highest CRP* (mg/L) | 69 (37 – 130) | 1 (1 – 4) | <0.0001 |
| sPLA-2 (pg/mL) | 87,231 (39,493 – 652,862) | 11,690 (6,496 – 21,609) | <0.0001 |

**Table S2. Characteristics of the sPLA-2/Ang LOS cohort.** Data are expressed as median (interquartile range 25^th^ - 75^th^ percentile) or n (%), as appropriate. *Within 72 hours of blood culture sampling. The Mann-Whitney test was used for continuous outcomes and Fisher’s exact test for categorical outcomes; *P* <0.05 was considered statistically significant.

|  | **Control**  **(n = 15)** | **Sepsis**  **(n = 15)** | ***p-value*** |
| --- | --- | --- | --- |
| Gestational Age (weeks) (median [IQR]) | 36.00  [33.50, 36.00] | 38.00  [31.00, 38.50] | 0.436 |
| Admission Weight (g)  (mean (SD)) | 1993.67 (535.29) | 2279.00 (766.81) | 0.247 |
| Birth Weight (g)  (mean (SD)) | 2125.33 (565.20) | 2370.00 (847.22) | 0.360 |
| Age (days)  (median [IQR]) | 2.00  [1.00, 3.00] | 3.00  [2.00, 3.00] | 0.142 |

**Table S3. Characteristics of infants from the Malawi cohort selected for qPCR analyses.** Data are expressed as median (interquartile range 25^th^ - 75^th^) or n (%), as appropriate. *Within 72 hours of blood culture sampling. *P* <0.05 considered statistically significant using Mann-Whitney test for continuous outcomes and Fisher’s exact test for categorical outcomes
